# The association of *FOXP3* gene polymorphisms with cancer susceptibility: a comprehensive systemic review and meta-analysis

**DOI:** 10.1042/BSR20181809

**Published:** 2019-03-19

**Authors:** Yan Chen, Xiaoxue Qi, Ce Bian, Chen Ling, Tao Yi, Xiyan Mu, Xia Zhao

**Affiliations:** 1 Department of Gynecology and Obstetrics, Key Laboratory of Obstetric and Gynecologic and Pediatric Diseases and Birth Defects of Ministry of Education, West China Second University Hospital, Sichuan University, Chengdu 610041, P.R. China; 2 Department of Gynecology and Obstetrics, Chengdu First People’s Hospital, Chengdu 610041, P.R. China

**Keywords:** cancer, FOXP3, meta-analysis, polymorphism, risk, susceptibility

## Abstract

The role of forkhead box P3 (FOXP3) protein in tumorigenesis has long been controversial and existing data on the association between *FOXP3* gene polymorphisms and cancer susceptibility were inconsistent. Here, we conducted a meta-analysis to better clarify the relationship. A comprehensive search of studies published from July 2008 to June 2018 was conducted. The statistical analyses of the pooled odds ratios (ORs) and the corresponding 95% confidence intervals (95% CIs) were performed using the Revman 5.2 software. A total of 12 articles with 19 case–control studies and 10389 participants were included. Three *FOXP3* polymorphisms and six cancer types were evaluated. While no significant results were observed in overall and breast cancer groups for rs3761548 (A/C) polymorphisms, the pooled data showed an elevated risk of cancer in variant AA genotypes and A allele for Chinese population (AA vs. AC+CC: OR = 1.61, 95% CI = 1.09, 2.39; AA vs. CC: OR = 1.74, 95% CI = 1.05, 2.89; A vs. C: OR = 1.34, 95% CI = 1.00, 1.78). Neither the overall group analyses nor the subgroup analyses stratified by cancer type and ethnicity proposed any significant association of rs2280883 (C/T) and rs3761549 (T/C) polymorphisms with cancer susceptibility. This meta-analysis suggested that *FOXP3* rs3761548 (A/C) polymorphisms were associated with increased cancer risk in Chinese population while rs2280883 (C/T) and rs3761549 (T/C) polymorphisms were not. More large-sample researches with diverse ethnicities and cancer types are needed to draw a concrete conclusion.

## Introduction

Tumor microenvironment (TME) plays an important role in cancer suppression and promotion [1]. As a crucial component of TME, regulatory T cells (Tregs) are responsible for down-regulating chronic inflammation, hindering autoimmune reactions, and maintaining peripheral immunological tolerance [2–4]. Recently published data demonstrated that Tregs-mediated immunosuppression was a pivotal tumor immune evasion mechanism, and might contribute to the failure of tumor immunotherapy [5,6]. A notable characteristic of Tregs is their expression of the transcription factor forkhead box P3 (FOXP3). The FOXP3 protein, which is encoded from *FOXP3* gene located on the X chromosome at Xp11.23, belongs to the forkhead/winged-helix transcription factor family and functions as a transcriptional repressor to down-regulate cytokine production of Tregs [7]. Several studies reported that FOXP3+ Tregs could infiltrate tumors at higher ratios than other T cells. The accumulation of FOXP3+Tregs in tumors and local lymph nodes could inhibit immune responses and thus result in tumorigenesis with a less favorable prognosis [8–10]. While Tregs are the major cell type expressing FOXP3 under physiological conditions, it has recently been found that FOXP3 was also expressed in a variety of cancers, such as ovarian, hepatocellular, pancreatic, and thyroid [11–14]. However, the role of FOXP3 as a tumor suppressor has also been documented. Zuo et al. [15] found that FOXP3 could be expressed in breast epithelial cells but was down-regulated in mammary cancer tissues. Li et al. [16] reported several mutations of this gene in prostate cancer patients and explored the tumor suppressor relationship between the FOXP3 and the Hippo pathways. This reminded us the complex role of FOXP3 and raised the possibility that mutations of *FOXP3* gene might cause immune dysregulation and further cancer development.

Genetic variants, mainly composed of single-nucleotide polymorphisms (SNPs), have been proved to cause alterations in protein function in multiple diseases [17]. Several *FOXP3* SNPs have been unveiled and their role in cancer susceptibility was explored. For example, Fazelzadeh Haghighi et al. [18] enrolled 312 Iranian participants and reported that T allele in rs3761549 (T/C) was correlated with susceptibility to lung cancer. You et al. [19] studied rs3761548 (A/C) polymorphisms in Chinese population and found the frequency of the A allele was significantly lower in endometrial cancer women than that in healthy controls. Another widely studied polymorphism was rs2280883 (C/T). Zheng et al. [20] recruited 1049 breast cancer patients from multiple centers in China but failed to report a significant correlation between allele C mutation and breast cancer risk.

To solve the controversy, a meta-analysis in 2014 was published and suggested that *FOXP3* rs3761549 (T/C) and rs3761548 (A/C) polymorphisms were not associated with the risk of breast cancer, but with the risk of lung cancer and hepatocellular cancer [21]. Unfortunately, it only included five articles with two types of polymorphisms. Since new case–control studies and more polymorphisms were published in recent years, we conducted a comprehensive search of relevant studies with the aim to better clarify the association between *FOXP3* polymorphisms and cancer susceptibility.

## Materials and methods

### Literature search

A comprehensive search of studies published from July 2008 to June 2018 was conducted in online databases of PubMed, Medline (Ovid), Embase, CNKI, Weipu, and Wanfang. The following search query was used: ‘FOXP3’, ‘Forkhead box protein’, ‘polymorphism’, ‘mutation’, ‘variant’, ‘cancer’, and ‘malignancy’. The search was updated twice a week until 30 June 2018. Language restrictions were not set and references of identified articles were also assessed for inclusion.

### Inclusion and exclusion criteria

An eligible study was included if it was consistent with the following criteria: (i) studied *FOXP3* polymorphisms in cancer risk; (ii) analyzed the polymorphisms that appeared in at least two independent articles for potential meta-analysis; (iii) provided sufficient data for extraction and calculation; and (iv) case–control studies based on human patients. When duplicated data appeared in different publications, only the most recent one was included. Meanwhile, studies that did not fulfill the above criteria were excluded.

### Data extraction and quality assessment

Potential studies were independently reviewed by two investigators (Y.C. and X.Q.). The following information was extracted from both cases and control groups: first author, year of publication, ethnicity, cancer type, SNPs, control type, genotyping method, adjusted parameters, and genotype distributions. Any discrepancies were resolved through a panel discussion until a consensus was reached.

The Newcastle–Ottawa Scale (NOS) was used to investigate the methodological quality of included studies. Three aspects of selection, comparability, and exposure were carefully evaluated. A score of 0–9 was determined and studies of moderate or high quality were included (score above 5). (http://www.ohri.ca/programs/clinical_epidemiology/oxford.asp) [22].

### Statistical analysis

To estimate the strength of the association between different *FOXP3* polymorphisms and cancer susceptibility, pooled odds ratios (ORs) and corresponding 95% confidence intervals (C95% Is) were calculated. Since SNPs were considered as binary variables of wild (W) and variant (V) alleles, five comparative models were used as follows: recessive model (VV vs. WW), dominant model (VV+ VW vs. WW), heterozygous model (VW vs. VV+WW), co-dominant model (VV+ VW vs. WW), and allelic model (V vs. W) [23]. Heterogeneity in each study was evaluated based on Higgins *I^2^* test. The random-effects model was applied when *I^2^* > 50%, indicating the presence of heterogeneity. Otherwise, if the *I^2^* was less than 50%, the fixed-effects model was used [24]. The Z-test was then performed to determine the significance of the pooled ORs where *P*<0.05 was illustrated as statistically significant. Eventually, the presence of publication bias was evaluated by visually inspecting the funnel plots. When asymmetry was suspected, Egger’s test was performed and *P_Egger_* above 0.05 indicating the absence of bias. All statistical analyses were performed using Revman 5.2 software (Cochrane Collaboration, Copenhagen, Denmark) except the Egger’s test, which was conducted using STATA 14.0 (StataCorp LP, College Station, TX, U.S.A.).

## Results

### Search results

**Figure 1:**
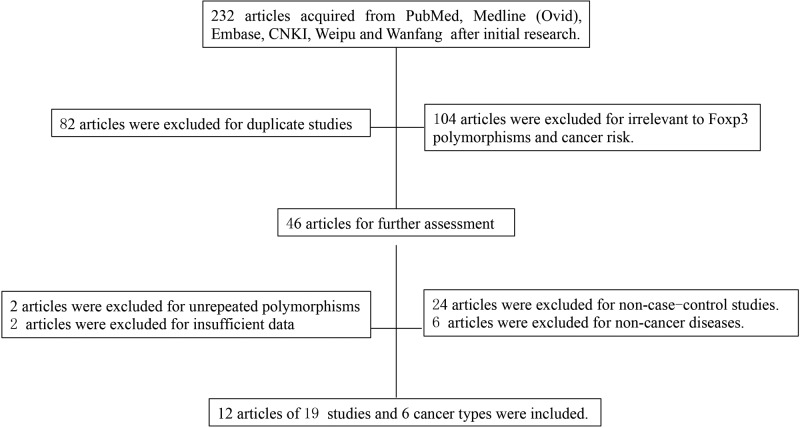
Flow chart of publication selection

As shown in Figure 1, a total of 232 publications were retrieved after the initial research. In our further screening, 82 articles were excluded for duplicity while 104 articles were removed since they were irrelevant to *FOXP3* polymorphisms or cancer risk according to titles and abstracts. Amongst the remaining 46 articles for full-text evaluation, 24 articles were biochemical studies, reviews, or meta-analysis; 6 articles studied non-cancer diseases such as autoimmune diseases; 2 articles analyzed several *FOXP3* polymorphisms that were not studied in other independent researches, resulting in the impossibility of data pooling; 2 articles failed to offer sufficient data for calculation. Therefore, we enrolled 12 articles in this meta-analysis [18–20,25–33].

### Characteristics of included studies

The 12 enrolled articles consisted of 19 case–control studies with three *FOXP3* polymorphisms (rs2280883 in four studies, rs3761548 in ten studies, and rs3761549 in five studies) and six cancer types (breast cancer in ten studies, colorectal cancer in one study, endometrial cancer in one study, hepatocellular in two studies, lung cancer in three studies, and thyroid cancer in two studies). Ethnicities included Brazilian, Chinese, Indian, Iranian, and Israeli. The control sources were population-based in two studies and hospital-based in eight studies (two studies failed to mention details on control type). Different genotyping methods were utilized including PCR-PAGE, PCR-restriction fragment length polymorphism (PCR-RFLP), and MS. The NOS showed that four articles were of high quality (NOS score of 8 or 9) and eight were of moderate quality (NOS score of 6 or 7). Adjusted parameters that might affect the cancer susceptibility were also listed (Table 1). All studies reported the numbers of corresponding genotypes for both case and control groups as to recessive, heterogeneous, and wild genotypes (Table 2).

**Table 1:** Characteristics of included studies

Authors	Year	Ethnicity	Cancer type	SNPs	Control type	Genotyping method	Adjusted parameters	Study quality (NOS)
Chen et al. [25]	2013	Chinese	Hepatocellular	rs2280883(C/T)	Hospital	MS	Age	8
				rs3761549(T/C)				
Chen et al. [26]	2014	Chinese	Colorectal	rs3761548(A/C)	Hospital	PCR-PAGE	Age, gender, smoking, alcohol drinking, family history of cancer	8
Fazelzadeh Haghighi et al. [18]	2015	Iranian	Lung	rs2280883(C/T)	Unknown	PCR-RFLP	Age, gender, family history of cancer	6
				rs3761549(T/C)				
He et al. [27]	2013	Chinese	Lung	rs3761548(A/C)	Hospital	PCR-PAGE	Age, gender	7
Banin Hirata et al. [28]	2017	Brazilian	Breast	rs3761548(A/C)	Unknown	PCR-RFLP	Age	7
Jahan et al. [29]	2014	Indian	Breast	rs3761548(A/C)	Hospital	PCR-RFLP	Age, menopausal status,	7
				rs3761549(T/C)			family history of cancer	
Jiang et al. [30]	2017	Chinese	Thyroid	rs2280883(C/T)	Hospital	PCR-RFLP	Age, gender	8
				rs3761548(A/C)				
Lopes et al. [31]	2014	Brazilian	Breast	rs3761548(A/C)	Population	PCR-RFLP	Age	8
Raskin et al. [32]	2009	Israeli	Breast	rs3761548(A/C)	Population	PCR-RFLP	Age	7
Tian et al. [33]	2018	Chinese	Breast	rs3761548(A/C)	Hospital	MS	Age, menopausal status, procreative	7
				rs3761549(T/C)			times	
You et al. [19]	2018	Chinese	Endometrial	rs3761548(A/C)	Hospital	PCR-RFLP	Age, BMI, family history of cancer, menopausal status, history of pregnancy	7
Zheng et al. [20]	2013	Chinese	Breast	rs2280883(C/T)	Hospital	MS	Age, BMI, family history of cancer,	7
				rs3761548(A/C)			age at menarche	
				rs3761549(T/C)				

Abbreviation: BMI, body mass index.

**Table 2:** Numbers of genotypes in cases and controls

SNPs	Authors	Year	Ethnicity	Cancer type	Case number	Control number	Case			Control		
							VV	VW	WW	VV	VW	WW
rs2280883 (C/T)	Chen et al. [25]	2013	Chinese	Hepatocellular	392	372	54	26	312	41	64	267
	Fazelzadeh Haghighi et al. [18]	2015	Iranian	Lung	30	30	1	14	15	4	13	13
	Jiang et al. [30]	2017	Chinese	Thyroid	350	306	13	49	288	10	69	227
	Zheng et al. [20]	2013	Chinese	Breast	1049	1091	35	365	649	31	349	711
rs3761548 (A/C)	Chen et al. [26]	2014	Chinese	Colorectal	360	400	57	123	180	29	114	257
	He et al. [27]	2013	Chinese	Lung	192	259	37	80	75	18	80	161
	Banin Hirata et al. [28]	2017	Brazilian	Breast	117	300	14	48	55	41	132	127
	Jahan et al. [29]	2014	Indian	Breast	202	130	27	160	15	20	106	4
	Jiang et al. [30]	2017	Chinese	Thyroid	350	306	19	109	222	11	73	222
	Lopes et al. [31]	2014	Brazilian	Breast	50	115	6	17	27	4	66	45
	Raskin et al. [32]	2009	Israeli	Breast	1444	1458	320	722	402	303	763	392
	Tian et al. [33]	2018	Chinese	Breast	559	581	24	198	337	20	173	388
	You et al. [19]	2018	Chinese	Endometrial	269	333	13	83	173	21	134	178
	Zheng et al. [20]	2013	Chinese	Breast	1049	1091	38	338	673	30	342	719
rs3761549 (T/C)	Chen et al. [25]	2013	Chinese	Hepatocellular	388	362	59	28	301	41	88	233
	Fazelzadeh Haghighi et al. [18]	2015	Iranian	Lung	30	30	1	4	25	0	3	27
	Jahan et al. [29]	2014	Indian	Breast	202	130	0	198	4	0	128	2
	Tian et al. [33]	2018	Chinese	Breast	560	582	18	157	385	23	187	372
	Zheng et al. [20]	2013	Chinese	Breast	1049	1091	32	283	734	34	290	767

### Quantitative analysis

**Figure 2:**
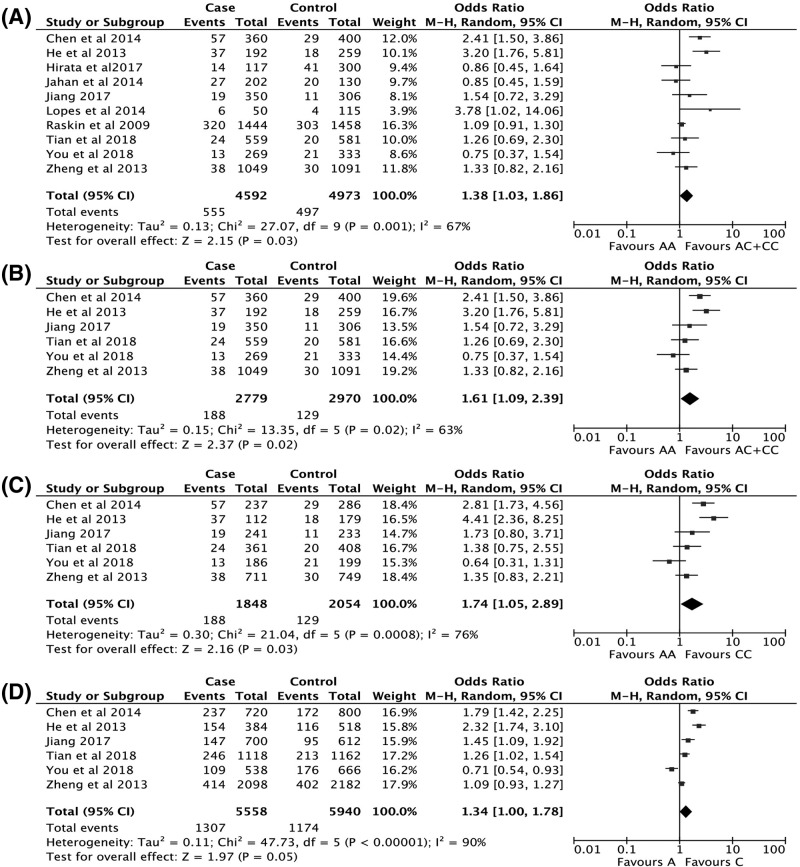
Representative forest plots of rs3761548 (A/C) polymorphisms and cancer susceptibility (**A**) AA vs. AC+CC in overall group analysis. (**B**) AA vs. AC+CC in Chinese group analysis. (**C**) AA vs. CC in Chinese group analysis. (**D**) A vs. C in Chinese group analysis. Abbreviations: df, degree of freedom; M–H, Mantel and Haenszel.

Pooled ORs and corresponding 95% CIs were shown in Table 3. Ten studies including 9565 participants were evaluated in rs3761548 (A/C) polymorphisms. For the overall group analysis, only one comparative model indicated an increased cancer risk while the remaining four failed to present any statistical significance (AA vs. AC+CC: OR = 1.38, 95% CI = 1.03, 1.86; AA+AC vs. CC: OR = 1.11, 95% CI = 0.87, 1.42; AC vs. AA+CC: OR = 1.02, 95% CI = 0.86, 1.23; AA vs. CC: OR = 1.39, 95% CI = 0.94, 2.05; A vs. C: OR = 1.16, 95% CI = 0.95, 1.40; Figure 2A). Thus it was impossible to draw a definite conclusion. Since six out of ten studies were focussed on breast cancer and Chinese population respectively, corresponding subgroup analyses were conducted. The results of 7096 participants showed no significant correlation between rs3761548 (A/C) polymorphisms and breast cancer susceptibility. However, an elevated risk in Chinese population was observed by using random-effects models in the enrolled 2779 cases and 2970 controls (AA vs. AC+CC: OR = 1.61, 95% CI = 1.09, 2.39; AA vs. CC: OR = 1.74, 95% CI = 1.05, 2.89; A vs. C: OR = 1.34, 95% CI = 1.00, 1.78; Figure 2B–D). It could be concluded that rs3761548 (A/C) polymorphism was associated with increased cancer risk in Chinese population.

**Table 3:** Summary of different comparative results of FOXP3 polymorphisms on cancer susceptibility

SNPs	Genotypes and alleles	Overall and subgroup	Participant number	OR [95% CI]	Z value	*P*-value	*I^2^* (%)	Effect model
rs2280883 (C/T)	CC vs. CT+TT	Overall	3620	1.18 [0.87, 1.59]	1.08	0.28	0	Fixed
		Chinese	3560	1.23 [0.91, 1.66]	1.33	0.18	0	Fixed
	CC+CT vs. TT	Overall	3620	0.79 [0.53, 1.18]	1.14	0.25	80	Random
		Chinese	3560	0.79 [0.50, 1.24]	1.01	0.31	86	Random
	CT vs. CC+TT	Overall	3620	0.69 [0.36, 1.30]	1.16	0.25	89	Random
		Chinese	3560	0.62 [0.29, 1.29]	1.29	0.20	93	Random
	CC vs. TT	Overall	2671	1.12 [0.83, 1.52]	0.74	0.46	0	Fixed
		Chinese	2638	1.15 [0.85, 1.57]	0.91	0.36	0	Fixed
	C vs. T	Overall	7240	0.88 [0.68, 1.14]	0.96	0.34	68	Random
		Chinese	7120	0.90 [0.68, 1.20]	0.72	0.47	76	Random
rs3761548 (A/C)	AA vs. AC+CC	Overall	9565	1.38 [1.03, 1.86]	2.15	0.03	67	Random
		Breast	7096	1.10 [0.95, 1.28]	1.30	0.19	8	Fixed
		Chinese	5749	1.61 [1.09, 2.39]	2.37	0.02	63	Random
	AA+AC vs. CC	Overall	9565	1.11 [0.87, 1.42]	0.83	0.41	84	Random
		Breast	7096	1.02 [0.92, 1.13]	0.44	0.66	60	Random
		Chinese	5749	1.35 [0.98, 1.86]	1.84	0.07	87	Random
	AC vs. AA+CC	Overall	9400	1.02 [0.86, 1.23]	0.27	0.79	72	Random
		Breast	7096	0.95 [0.78, 1.16]	0.51	0.61	64	Random
		Chinese	5749	1.17 [0.94, 1.45]	1.40	0.01	71	Random
	AA vs. CC	Overall	5704	1.39 [0.94, 2.05]	1.65	0.10	78	Random
		Breast	3031	1.06 [0.89, 1.26]	0.69	0.49	28	Fixed
		Chinese	3,902	1.74 [1.05, 2.89]	2.16	0.03	76	Random
	A vs. C	Overall	19130	1.16 [0.95, 1.40]	1.63	0.10	88	Random
		Breast	13862	1.04 [0.96, 1.12]	0.95	0.34	26	Fixed
		Chinese	11498	1.34 [1.00, 1.78]	1.97	0.05	90	Random
rs3761549 (T/C)	TT vs. TC+CC	Overall	4424	1.12 [0.84, 1.48]	0.77	0.44	0	Fixed
		Breast	3614	0.91 [0.62, 1.34]	0.48	0.63	0	Fixed
		Chinese	4032	1.11 [0.83, 1.47]	0.70	0.49	17	Fixed
	TT+TC vs. CC	Overall	4424	0.80 [0.58, 1.10]	1.37	0.17	70	Random
		Breast	3614	0.93 [0.80, 1.08]	0.93	0.35	11	Fixed
		Chinese	4032	0.77 [0.54, 1.10]	1.45	0.15	84	Random
	TC vs. TT+CC	Overall	4424	0.67 [0.38, 1.18]	1.38	0.17	88	Random
		Breast	3614	0.94 [0.81, 1.10]	0.77	0.44	0	Fixed
		Chinese	4032	0.61 [0.32, 1.17]	1.50	0.13	94	Random
	TT vs. CC	Overall	3058	1.00 [0.75, 1.33]	0.03	0.97	0	Fixed
		Breast	2371	0.89 [0.60, 1.31]	0.59	0.56	0	Fixed
		Chinese	3005	0.98 [0.74, 1.31]	0.11	0.91	0	Fixed
	T vs. C	Overall	8848	0.91 [0.82, 1.02]	1.68	0.09	34	Fixed
		Breast	7228	0.95 [0.84, 1.07]	0.90	0.37	6	Fixed
		Chinese	8064	0.88 [0.74, 1.04]	1.48	0.14	54	Random

The meta-analysis of the other two polymorphisms failed to show significant association between variant genotypes (or alleles) and cancer susceptibility in corresponding effect models. Briefly, for rs2280883 (C/T) polymorphisms, 3620 participants including 1821 cases and 1799 controls were analyzed. None of the five comparative models displayed any relationship between rs2280883 (C/T) polymorphisms and cancer risk, neither in the overall group analysis (CC vs. CT+TT: OR = 1.18, 95% CI = 0.87, 1.59; CC+CT vs. TT: OR = 0.79, 95% CI = 0.53, 1.18; CT vs. CC+TT: OR = 0.69, 95% CI = 0.36, 1.30; CC vs. TT: OR = 1.12, 95% CI = 0.83, 1.52; C vs. T: OR = 0.88, 95% CI = 0.68, 1.14) nor in the stratified Chinese group analysis (CC vs. CT+TT: OR = 1.23, 95% CI = 0.91, 1.66; CC+CT vs. TT: OR = 0.79, 95% CI = 0.50, 1.24; CT vs. CC+TT: OR = 0.62, 95% CI = 0.29, 1.29; CC vs. TT: OR = 1.15, 95% CI = 0.85, 1.57; C vs. T: OR = 0.90, 95% CI = 0.68, 1.20). Amongst the five studies that focussed on rs3761549 (T/C) polymorphisms, three reported increased cancer risk for mutated genotypes while two reported insignificant results. By pooling the 4424 participants together, no significant correlation was found between T/C mutation and cancer risk. The results of subgroup analysis on breast cancer and Chinese population were consistent with the overall group analysis.

### Publication bias

**Figure 3:**
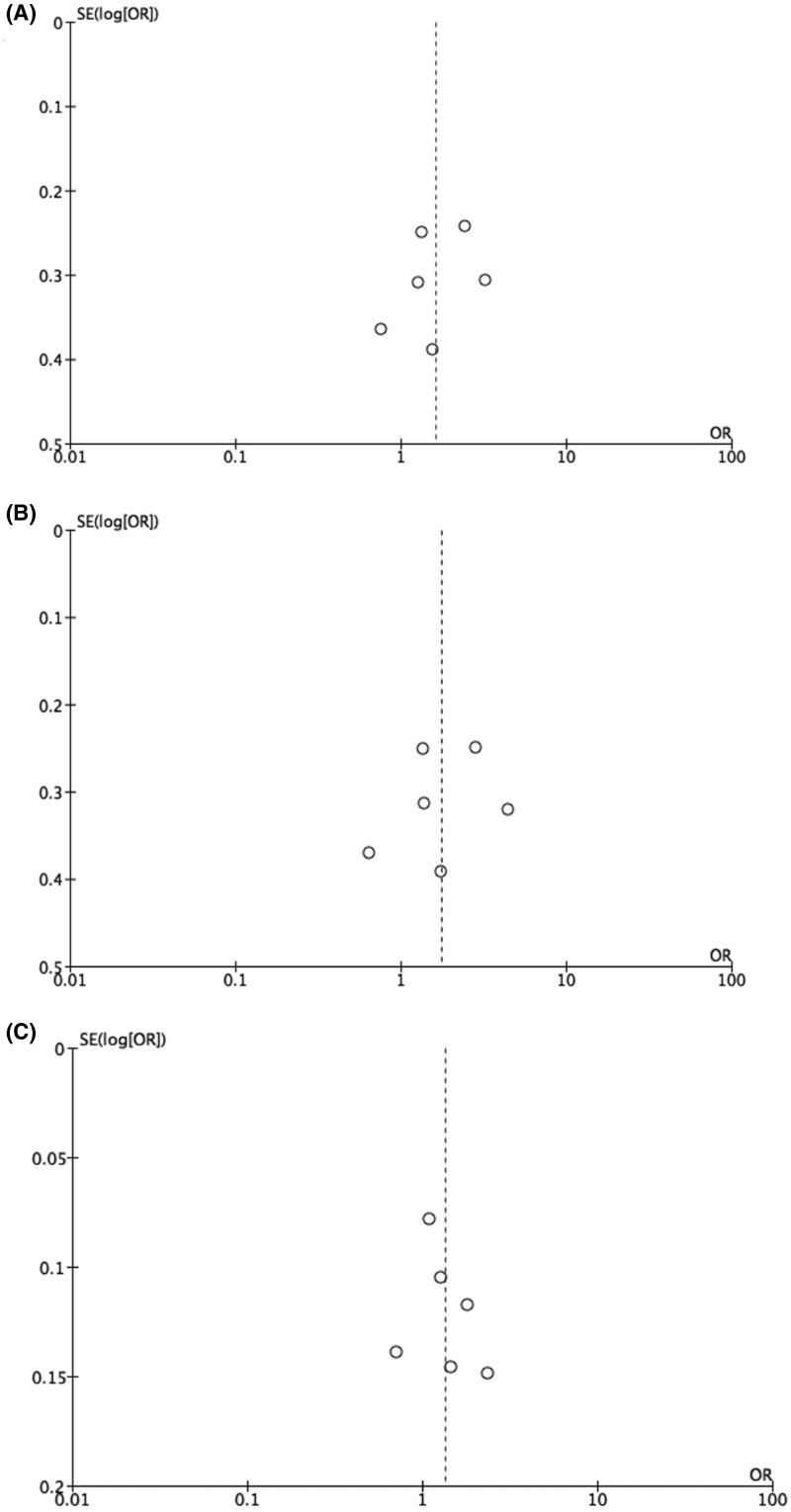
Representative funnel plots of publication bias of rs3761548 (A/C) polymorphisms and cancer susceptibility (**A**) AA vs. AC+CC in Chinese group analysis. (**B**) AA vs. CC in Chinese group analysis. (**C**) A vs. C in Chinese group analysis. Abbreviation: SE, standard error.

The publication bias was visually examined on the funnel plots generated by Revman 5.2 software. No obvious asymmetry could be observed (Figure 3). We further performed Egger’s tests in the three analyses that proposed significant association between rs3761548 (A/C) polymorphisms and cancer susceptibility in Chinese population. The results demonstrated no significant publication bias (*P*>0.05, data not shown).

## Discussion

Since alteration of the human immune system can contribute to the development of human cancer, FOXP3 has attracted attention in recent decades as one of the main transcription factors for Tregs, an important participant of immune evasion and surveillance in TME [34–36]. While imbalance of FOXP3+Tregs has been widely reported in autoimmune diseases such as allergic rhinitis and Graves’ disease, the role of FOXP3 in tumorigenesis has long been controversial [37,38]. FOXP3 is able to repress oncogenes while activating additional tumor suppressor genes. Evidences of this dual role include the down-regulation of *MYC* and *HER2* by FOXP3+ Tregs, and the up-regulation of FOXP3 protein in both Tregs and tumor cells in patients with lung cancer and hepatocellular carcinoma [39–42]. Polymorphisms of the *FOXP3* gene may change FOXP3 protein quantitatively or functionally, thus contributing to predisposition and progression of cancer. To date, several polymorphisms of *FOXP3* have been found including rs2280883, rs3761548, rs3761549, rs2294020, rs2294021, rs5906761, rs5902434 etc [19,20,32]. Their roles in cancer susceptibility remain undetermined due to sample size, ethnicity, and other confounding factors. Here, we conducted a thorough meta-analysis with the aim to address the inconsistencies of existing publications and to draw a more concrete conclusion.

We enrolled 19 case–control studies with 10389 participants in this meta-analysis. Three types of *FOXP3* polymorphisms and six types of cancers were analyzed. The results showed that rs3761548 (A/C) were correlated with cancer susceptibility in Chinese population. The variant AA genotypes and A allele imposed a significant higher cancer risk compared with their counterparts (AA vs. AC+CC: OR = 1.61, 95% CI = 1.09, 2.39; AA vs. CC: OR = 1.74, 95% CI = 1.05, 2.89; A vs. C: OR = 1.34, 95% CI = 1.00, 1.78). Notably, while the breast subgroup analysis failed to present any significance, only one comparative model in the overall group suggested that AA genotypes proposed a 1.38-fold increased risk compared with AC plus CC genotypes (OR = 1.38, 95% CI = 1.03, 1.86). The reason behind this was due to the Lopes et al.’s [31] study of triple-negative breast cancer (TNBC) in Brazil which suggested that AC heterozygous genotype was a protective factor while AA was a risky one. If we excluded it from the meta-analysis, no significant results could be drawn (AA vs. AC+CC: OR = 1.33, 95% CI = 0.99, 1.78). Interestingly, in the article published by You et al. [19], both the variant A allele and mutated genotypes (AA plus AC) in rs3761548 showed a statistically significant protective effect on endometrial cancer, which is contrary to the rest of included studies [19]. If we excluded it from the meta-analysis, an elevated risk was again concluded for A/C mutation in overall cancer risk (AA vs. AC+CC: OR = 1.47, 95% CI = 1.07, 2.00). The intriguing results reminded us the controversial role of *FOXP3* in cancer immunity, especially in different cancer types. As an X-linked gene, the mutated *FOXP3* in females depends on X-chromosome inactivation [43,44]. Whether this rs3761548 position was related to gender-specific cancers or hormone-related cancers such as breast and endometrial cancers remained to be solved. Another relevant point lies in the location of mutated FOXP3 protein in different tumor cells. According to Lopes et al. [31], most TNBC patients had cytoplasmic expression of FOXP3 protein and only some had concomitant perinuclear and/or nuclear expression. The re-localization of FOXP3 protein due to polymorphisms like rs3761548 in certain types of cancer might affect transcription functions and thus cytokine production of Tregs [45].

The results of rs3761549 (T/C) polymorphisms in cancer susceptibility were consistent with the previous meta-analysis [21]. With 4424 participants enrolled, the meta-analysis revealed no significant relationship between rs3761549 (T/C) polymorphisms and cancer risk. However, whether T/C mutations were correlated with increased risk of hepatocellular cancer like previously described remained questionable due to non-repeated researches and limited sample sizes. Specifically, our subgroup analysis stratified by Chinese population indicated a lack of significant association, which was never reported before. Like rs3761548 (A/C), rs3761549 (T/C) polymorphisms were also located in the promoter region of the *FOXP3* gene, which is considered to cause mRNA instability thus affecting FOXP3 production and activity [46,47]. The specific reason why the two types of polymorphisms acted differently in case–control studies is not clear, which is a promising subject for future studies. We also explored the role of rs2280883 (C/T) polymorphisms in cancer risk. The meta-analysis failed to draw a significant conclusion, neither in general sample nor in different subtypes. The less aggressive role of rs2280883 (C/T) polymorphisms might be due to the location, which was in intron 9 very near a conserved transcription region of *FOXP3* gene. This could cause splicing downstream, resulting in a less functional gene. Further researches are needed to consolidate the exact mechanism [30,48].

Despite our efforts to include all the existing publications, some disadvantages of the present meta-analysis should be notified. First, insufficient published studies were enrolled in this meta-analysis. Although the number of participants was so far the largest, more individual studies were still required to determine a precise conclusion, especially for rs2280883 (C/T) polymorphisms. Second, amongst the 19 studies, only six types of cancer were included. It is known that Tregs play a dual role in tumorigenesis. Whether the tumor promotion effect takes place or the contrary is largely due to the biological characteristics of primary cancer [49]. Thus, caution must be paid when explaining the results to other cancers such as the sex-related and hormone-related cancers. Third, the populations of included studies were Chinese, Iranian, Brazilian, Israeli, and Indian, and many other ethnicities like blacks and Caucasians were ignored. This might affect the overall group analysis since we noticed a different result when Chinese population was stratified for rs3761548 (A/C) polymorphisms. Fourth, our evaluation was based on unadjusted results. Risk factors like body mass index, smoking habit, and menstruation status are also known to be important to tumorigenesis in several types of cancer [50,51]. These confounding factors might cause distorted results. Therefore, studies on more types of cancer are needed to help draw a concrete conclusion.

Taken together, based on current articles in databases, our meta-analysis suggested that *FOXP3* rs3761548 (A/C) polymorphisms were associated with cancer risk in Chinese population while no significant correlation was confirmed in rs2280883 (C/T) and rs3761549 (T/C) polymorphisms. To the best of our knowledge, the present study was the most comprehensive one to explore the relationship between *FOXP3* polymorphisms and cancer risk. It is also the first to pool the results of rs2280883 (C/T) polymorphisms and conduct subgroup analysis stratified by ethnicity. Considering the limitations mentioned above, more large-sample researches with diverse ethnicities and cancer types are needed to help reach a consensus.

## Abbreviations

95% CI: confidence interval
FOXP3: forkhead box P3
NOS: Newcastle–Ottawa Scale
OR: odds ratio
SNP: single-nucleotide polymorphism
TME: tumor microenvironment
TNBC: triple-negative breast cancer
Treg: regulatory T cell
